# Long-term outcome of vaginal mesh or native tissue in recurrent prolapse: a randomized controlled trial

**DOI:** 10.1007/s00192-017-3512-3

**Published:** 2017-11-22

**Authors:** Alfredo L. Milani, Anne Damoiseaux, Joanna IntHout, Kirsten B. Kluivers, Mariella I. J. Withagen

**Affiliations:** 1 Department of Obstetrics & Gynecology, Reinier de Graaf Hospital, P.O. Box 5011, 2600 GA Delft, The Netherlands; 2 Department of Obstetrics & Gynecology, Catharina Hospital, P.O. Box 1350, 5602 ZA Eindhoven, The Netherlands; 3 Department for Health Evidence (133), Radboud Institute for Health Sciences, Radboud University Medical Center, P.O. Box 9101, 6500 HB Nijmegen, The Netherlands; 4 Department of Obstetrics & Gynecology (791), Radboud University Medical Center, P.O. Box 9101, 6500 HB Nijmegen, The Netherlands; 5 Department of Obstetrics & Gynecology, University Medical Center Utrecht, Heidelberglaan 100 P.O. Box 85500, Room F05.126, Utrecht, The Netherlands

**Keywords:** Long-term outcome, Mesh, Native tissue, Pain, Pelvic organ prolapse, Surgery

## Abstract

**Introduction and hypothesis:**

Our aim was to evaluate clinically relevant long-term outcomes of transvaginal mesh or native tissue repair in women with recurrent pelvic organ prolapse (POP).

**Methods:**

We performed a 7-year follow-up of a randomized controlled trial on trocar-guided mesh placement or native tissue repair in women with recurrent POP. Primary outcome was composite success, defined as absence of POP beyond the hymen, absence of bulge symptoms, and absence of retreatment for POP. Secondary outcomes were adverse events, pain, and dyspareunia. Multiple imputation was used for missing data of composite success and pain; estimates are presented with 95% confidence intervals (CI).

**Results:**

Between August 2006 and July 2008, 194 women were randomized; 190 underwent surgery. At 7 years, 142 (75%) were available for analysis, of whom, the primary outcome could be calculated in 127. Composite success was 53% (95% CI 41, 66) for mesh and 54% (95% CI 42, 65) for native tissue. Repeat surgery for POP was 25% for mesh and 16% for native tissue (difference 9%; 95% CI −5, 23) and occurred in untreated compartments in the mesh group and treated compartments in the native tissue group. Mesh exposure rate was 42%; pain with mesh 39% and native tissue 50% (difference − 11%, 95% CI −27, 6); dyspareunia with mesh 20% and native tissue 17% (difference 3%, 95% CI −9, 17).

**Conclusions:**

Seven-year composite success rates appeared similar for mesh and native tissue. Mesh did not reduce long-term repeat surgery rates due to de novo POP in nonmesh-treated vaginal compartments. Mesh exposure rates were high, though significant differences in pain and dyspareunia were not detected.

**Clinical trial registration**. ClinicalTrials.gov, NCT00372190.

## Introduction

The 10-year rate of repeat surgery for pelvic organ prolapse (POP) and urinary incontinence (UI) is as high as 17% [1]. Searching for more effective treatments and inspired by favorable results of synthetic mesh use in inguinal hernia and stress urinary incontinence (SUI) surgery, mesh was introduced in vaginal POP surgery [2–4]. The commercial introduction of the relatively easy to use mesh kit in 2005 has accelerated worldwide use of synthetic mesh, while 1-year outcomes of the first prospective observational study were first published in 2007 [5]. An increasing number of adverse events, including mesh exposure, pain, and dyspareunia led to the US Food and Drug Association (FDA) Public Health Notifications of 2008 and 2011, with the intention to increase public awareness of risks associated with surgical mesh for transvaginal repair of POP [6]. Subsequent class action lawsuits occurred and gradually led to removal of many vaginal mesh products from the market.

A limited number of randomized clinical trials (RCT) compared short-term safety and efficacy of nonabsorbable mesh with native tissue repair [7]. Long-term data of those trials are lacking, though sorely needed. This RCT focused on patients with recurrent POP and compared a trocar-guided nonabsorbable tension-free vaginal mesh (TVM) with native tissue repair [8]. At 12 months, anatomic failures after TVM appeared fewer than native tissue repair, but symptom decrease and quality of life (QoL) improvement were similar between groups [8].

The study reported focused on the long-term follow-up of that RCT, with the primary aim being comparison of long-term composite success (a combination of anatomy, functional success, and absence of retreatment) 7 years after surgery. The secondary aim was to describe long-term adverse events, with particular focus on pain and dyspareunia.

## Materials and methods

The original trial was performed in 13 centers in The Netherlands between August 2006 and July 2008 (www.clinicaltrials.gov, NCT00372190). Patients with recurrent POP were 1:1 randomized between a first-generation nonabsorbable TVM (anterior, posterior or total) (Prolift™, Ethicon, Somerville, NJ, USA) and conventional vaginal native tissue repair. Repairs comprised anterior or posterior colporrhaphies; a Manchester Fothergill procedure; or vaginal hysterectomy with high uterosacral ligament suspension, sacrospinous ligament suspension, or a combination. Details regarding design, randomization, sample size, surgical interventions, and 1-year outcomes have previously been published [8]. Extended follow-up at 7 and 10 years was approved by the Medical Ethics Committee, region Arnhem-Nijmegen, The Netherlands, on 28 April 2014 under no. NL46834.091.14 and registered in clinicalTrials.gov, NCT00372190. Informed consent was obtained prior to inclusion in the extended follow-up.

All participants of the index study received a letter to inform them on the intended long-term follow-up. Two weeks later, a research nurse approached those women by telephone and scheduled an outpatient clinic appointment if they agreed to participate. One independent examiner (AD), a subspecialist in urogynecology, was blinded to the index procedures and performed all interviews and physical examinations at the original study sites. Participants completed the same validated urogynecologic questionnaires as were used at baseline and 1 year in the index study: Global Impression of Improvement questionnaire (PGI-I), visual analog scale (VAS), EuroQol-5D (EQ-5D), Urogenital Distress Inventory (UDI), Defecatory Distress Inventory (DDI), Incontinence Impact Questionnaire (IIQ), and the short form of the Pelvic Organ Prolapse Urinary Incontinence Sexual Questionnaire (PISQ-12) [9–12]. The UDI, DDI, and IIQ are each subdivided into five domains, with subscales ranging from 0 to 100 and higher scores indicating more bother and worse QoL. Scores on 12 individual questions of the PISQ-12 ranged from 0 to 4 on a 5-point Likert scale, and total scores range from 0 (poorest) to 48 (best) for sexual function [13].

Subjective prolapse symptoms were considered present if a patient answered affirmatively to the question: Do you see or do you feel a vaginal bulge? Pain was considered present if a patient answered affirmatively to the question: Do you experience pain in the lower abdomen or genital region? Dyspareunia was considered present if a patient answered affirmatively to the question: Do you experience pain during intercourse? The degree of bother caused by dyspareunia was registered using a 4-point Likert scale (1, not at all bothered; 4 bothered quite a bit). SUI was considered present if a patient answered affirmatively to the question: Do you experience involuntary urine loss during physical exercise, coughing, or sneezing? De novo SUI was considered present if the participant had no SUI according the baseline questionnaire but answered affirmatively at 7 years or had received treatment for SUI after the trial’s index surgery within the 7-year follow-up.

Pain was assessed with a 10-point VAS, where 0 denoted no pain and 10 the worst imaginable. Prior to physical examination, women were requested to undergo two VAS for pain: one at rest and one during physical activity.

Physical examination consisted of POP staging using the Pelvic Organ Prolapse Quantification (POP-Q) system [14]. A thorough examination of the vagina was performed to objectify any visible or palpable mesh exposure, prominence, or excessive scarring of the vaginal epithelium and pain during vaginal examination [15]. Participants were asked to rate pain intensity during vaginal examination on another VAS. The examiner also completed a VAS of subjective impression she had of pain experienced by the patient, blinded to the patient’s score, and was not informed on this specific assessment prior to the examination.

In contrast with the primary outcome at 1 year, defined as an anatomic failure in treated vaginal compartments, the primary outcome of the study reported here was composite success, defined as a combination of absence of POP beyond the hymen in a treated or nontreated compartment, absence of bulge symptoms, and absence of retreatment for POP in a treated or nontreated compartment [16]. If no POP beyond the hymen was noted on POP-Q examination but the questionnaire was missing, a composite outcome could not be calculated. This also applied if the questionnaire noted absence of bulge symptoms, but the POP-Q was missing. If, however, the questionnaire noted bulge symptoms, but the POP-Q was missing, the composite outcome was considered a failure, as was POP beyond the hymen without a questionnaire. Any registered retreatment for POP, with or without follow-up, was considered a failure. Retrospectively, composite success rates were calculated for all participants at the 1-year follow-up and used for comparison over time.

Secondary outcomes were anatomic recurrences overall and failures per treated vaginal compartment at 7 years, defined as POP stage ≥ II, POP > hymen, de novo SUI, surgery-related complications for mesh, pain perception, and dyspareunia. Descriptive statistics with numbers, percentages, and risk differences with 95% CI were used to summarize baseline characteristics and primary and secondary outcomes. The 95% CI for differences between median values was estimated with the Hodges–Lehmann estimator [17].

To account for missing data of women who were alive but did not attend the 7-year follow-up visit, a multiple imputation approach was used for composite success and UDI domain of pain. Analyses were performed using the combination of 50 data sets generated with the fully conditional method (chained equations) for imputation of missing values. Values imputed for pain <0 or >100 were truncated to 0 or 100, and composite success was imputed as a binary variable. Missing data of women who died during the 7-year follow-up were not imputed in the main analysis, but were imputed as failures in a sensitivity analysis of composite success. After multiple imputation, composite success was analyzed using a binomial distribution, an identity link, and covariate treatment group. UDI domain pain was analyzed using a linear model, with covariate treatment group and pain at baseline.

Analysis was according the intention-to-treat principle. Statistical analyses were performed using SPSS Statistics for Windows, version 22.0 and analyses of pain and composite success with the STAT package (SAS® statistical software, version 9.4, SAS Institute Inc., Cary, NC, USA).

## Results

**Fig. 1:**
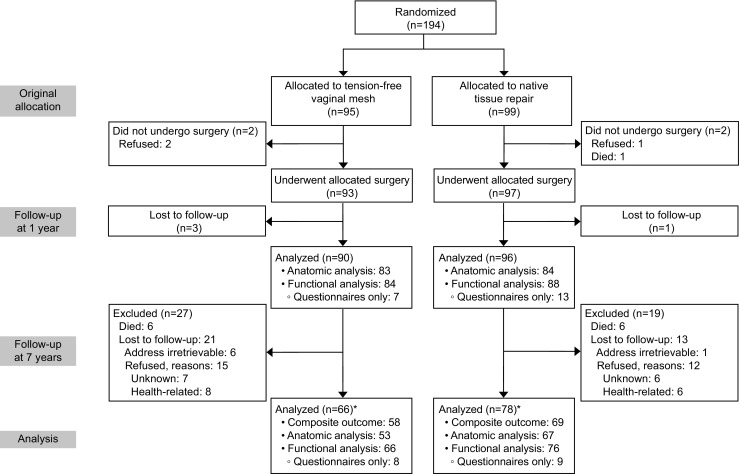
Consolidated Standards of Reporting Trials (CONSORT) flowchart of randomization and follow-up. *Includes those lost to follow-up at 1 year

One hundred and ninety-four women were randomized in the index trial between August 2006 and July 2008, of whom 190 underwent surgical treatment. In this 7-year follow-up, 142 (75%) women were available for analysis (Fig. 1). Based on the selection criteria described in the Materials and Methods section, a composite outcome could be calculated of 127 participants. Of 15 women data were incomplete, so a composite outcome could not be calculated.

Characteristics of participants in the 7-year follow-up were similar between groups at baseline (Table 1), including post hoc calculated composite success rates at 1 year (76%, 95% CI 62, 85 vs. 74%, 95% CI 62, 84 for mesh and native tissue, respectively). Some differences existed between responders and nonresponders in the study reported here: responders were significantly younger and had lower POP stages at the time of index surgery and more often had reported dyspareunia at baseline. General health scores (VAS, EQ-5D) at 1-year were higher among responders (Table 2).

**Table 1:** Characteristics of responders (participants in the 7-year follow-up) by treatment group

	Mesh (*n* = 58)	Native tissue (*n* = 69)	Difference (95% CI)
Age at time of surgery (years)	60.9 ± 9.0	62.4 ± 10.2	−1.5 (−5.1, 1.7)
Age at follow-up (years)	67.1 ± 8.9	68.8 ± 9.9	−1.7 (−5.1, 1.7)
Follow-up (months)	84 (65–98)	84 (65–99)	0 (−3.0, 3.0)
Parity (*n*)	2 (1–6)	2 (1–5)	0 (0, 0)
BMI (kg/m^2^)	26.4 ± 4.1	26.8 ± 4.3	−0.4 (−2.0, 1.1)
Prior number of POP surgeries	1 (1–3)	1 (1–3)	0 (0, 0)
Prior number of treated vaginal compartments	2 (1–3)	2 (1–3)	0 (0, 0)
POP-Q stage at time of surgery			
• Stage II	33 (57)	36 (52)	5 (−13, 22)
• Stage III	24 (41)	31 (45)	−4 (−21, 14)
• Stage IV	1 (2)	2 (3)	−1 (−6, 4)
Pain at baseline			
• Yes	26/55 (47)	35/58 (60)	−13 (−31, 5)
• UDI pain score	26.1 (24.2, 28.0)	28.8 (26.3, 31.3)	−2.6 (−10.9, 5.6)
Dyspareunia at baseline			
• Moderate to quite a bit	11/53 (21)	15/58 (26)	−5 (−21, 11)
• Yes	18/53 (34)	27/58(46)	−12 (−31, 6)
Composite success and pain score at 1 year			
• Composite success;* n* [%: (95% CI)]	40/53 [76: (62, 85)]	43/58 [74: (62, 84)]	2 (−15, 18)
• UDI Pain score;* n* (CI)	13.2 (11.4, 15.0)	15.1 (13.5, 16.7)	−1.8 (−8.5, 4.9)

Data presented as mean ±  standard deviation (SD), median (range),* n* (%), I [%: (95% CI)], % (95% CI), or mean (95% CI)

*BMI* body mass index,* POP-Q* Pelvic Organ Prolapse Quantification system,* UDI pain score* urodynamic pain score, 0–100,* CI* confidence interval

**Table 2:** Characteristics of responders versus nonresponders (women lost to follow-up)

	Responders (*n* 127)	Lost to follow-up (*n* 63)	Difference (95% CI)
Age at time of surgery (years)	61.6 ± 9.7	67.7 ± 10.4	−6.1* (−9.2, −3.1)
Parity	2 (1–6)	3 (0–5)	0 (0, 1.0)
BMI (kg/m^2^)	26.6 ± 4.2	26.9 ± 4.1	−0.3 (−1.6, 1.1)
Prior number of POP surgeries	1 (1–3)	1 (1–2)	0 (0, 0)
Prior number of treated vaginal compartments	2 (1–3)	2 (1–3)	0 (0, 0)
POP-Q stage at time of surgery			
• Stage II	69 (54.3)	21 (33.3)	21.0* (6.5, 35.5)
• Stage III	55 (43.3)	42 (66.7)	−23.4* (−37.8, −8.9)
• Stage IV	3 (2.4)	0	2.4 (−0.3, 5.0)
Pain at baseline			
• Yes	61/113 (54.0)	23/48 (47.9)	6.1 (−10.8, 22.9)
• UDI pain score	28.7 (26.9, 30.4)	23.4 (19.8, 27.0)	5.3 (−3.7, 14.3)
Dyspareunia at baseline			
• Moderately to quite a bit	26/111 (23.4)	3/45 (6.7)	16.8* (6.0, 27.5)
• Yes	45/111 (40.5)	4/45 (8.9)	31.7* (19.3, 44.0)
Outcomes at 1-year follow-up			
Composite success	83/111 (74.8)	31/43 (72.1)	2.6 (−12.9, 18.3)
Overall POP-Q ≥ stage II	66/115 (57.4)	23/52 (44.2)	13.2 (−3.1, 29.4)
PGI-I (much to very much better)	78/108 (72.2)	31/49 (63.3)	8.9 (−6.9, 24.9)
VAS EQ-5D	78.2 ± 14.6	72.4 ± 20.3	5.8* (0.2, 11.5)
Exposure	9/114 (7.9)	5/52 (9.6)	−1.7 (−11.1, 7.7)
UDI pain score	14.3 (13.1, 15.5)	13.8 (10.4, 17.1)	0.5 (−6.9, 7.9)
Dyspareunia			
• Moderately to quite a bit	18/115 (16.2)	3/47 (4.7)	9.3 (−0.4, 18.9)
• Yes	35/115 (30.4)	8/46 (17.4)	13.0 (−0.8, 26.9)

Data presented as mean ±  standard deviation (SD), median (range),* n* (%), or mean (95% CI)

*BMI* body mass index, *PGI-I* Patient Global Impression of Improvement,* VAS EQ-5D* perceived health condition according visual analog scale, EuroQuol-5D questionnaire (100 = best imaginable, 0 = worst imaginable), * UDI pain score* urodynamic pain score, 0–100,* CI* confidence interval

*This difference would be statistically significant if tested at a significance level of 0.05

The primary outcome, overall composite success at 7 years, was similar between women treated with mesh or native tissue (53%; 95% CI 41, 66 vs. 54%; 95% CI 42, 65; Table 3). These results were similar to the percentages estimated after applying multiple imputations for missing data. Success rates were slightly lower after imputing failures for missing data of deceased patients, but the difference between groups remained negligible. Between 1 and 7 years, composite success rates decreased by 22% for mesh and 20% for native tissue (Table 3).

**Table 3:** Primary and secondary outcomes at 7 years

	Mesh	Native tissue	Risk difference (95% CI)
Primary outcome			
Composite success overall			
• Participants,* n* [% (95% CI)]	31/58 [53 (41, 66)]	37/69 [54 (42, 65)]	−1 (−18, 17)
• Estimated % (95% CI) after MI^a^	53 (40, 66)	54 (43, 65)	−1 (−18, 16)
• Estimated % (95% CI) after MI^b^	51 (38, 64)	50 (39, 61)	−1 (−17, 16)
Change in composite success between 1 and 7 years			
% (95% CI) of 7-year participant data	−22 (−39, −5)	−20 (−37, −4)	2 (−12, 16)
Composite failures	27/58 (47)	32/69 (46)	1 (−17, 18)
• Failure in treated compartment	3/58 (5)	18/69 (26)	−21* (−33, −9)
• Failure in untreated compartment	17/58 (29)	5/69 (7)	22* (9, 35)
• Failure due to bulge but no POP > hymen	5/58 (9)	1/69 (1)	7 (−1, 15)
• Failure due to bulge (questionnaire only)	2/58 (3)	8/69 (12)	−8 (−17, 1)
Secondary outcomes			
Reoperation for POP	14^c^/56 (25)	11^d^/69 (16)	9 (−5, 23)
• In treated compartment	1 (7)	9 (82)	−75* (−100, −48)
• In nontreated compartment	13 (93)	2 (18)	75* (48, 100)
POP ≥ stage II	28/53 (53)	47/67 (70)	−17 (−35, 0)
Reoperation for POP and/or POP-Q ≥ stage II	35/56 (62)	53/69 (77)	−15 (−30, 2)
POP > hymen	8/53 (15)	12/67 (18)	−3 (−16, 10)
POP > hymen, no sensation of bulge	4/53 (8)	7/67 (10)	−3 (−13,7)
Reoperation for POP and/or POP > hymen	22/58 (38)	23/69 (33)	5 (−12, 21)
Subjective outcomes			
• Sensation of bulge	14/66 (21)	17/76 (22)	−1 (−14,19)
• PGI-I (much to very much better)	42/60 (70)	41/72 (57)	13 (−3, 29)

Data presented as* n* (%),* n* [% (95% CI)], or % (95% CI)

*POP* pelvic organ prolapse,* PGI-I* Patient Global Impression of Improvement,* MI* multiple imputation of missing data,

^a^ MI excluding deceased patients

^b^ MI with failures for missings of deceased patients

^c^ Nine patients received another mesh

^d^ Nine patients received mesh

*This difference would be statistically significant if tested at a significance level of 0.05

Repeat surgery rates for POP were somewhat different between groups (Table 3). In the mesh group, repeat surgery was performed in 14/56 cases. Nine times an additional mesh was inserted (8 vaginally and 1 abdominally), and in 13 cases, surgery was in the non-mesh-treated compartment. In contrast, repeat surgery in the native tissue group occurred in 11/69 cases, and more frequently in the treated vaginal compartment. Nine times a mesh was inserted in this group (8 vaginally and 1 abdominally). In the mesh group, there were fewer anatomic failures (POP ≥ II) in the treated anterior vaginal compartment compared with in the native tissue group. Bulge symptoms and patient global impressions of improvement were not significantly different between groups (Table 7, “Appendix 3”).

At 7 years, there was no significant difference between groups in terms of de novo SUI (19% mesh, 12% native tissue) (Table 8 "Appendix 4"). The cumulative prevalence of mesh exposure at 7 years was 42% in the mesh group, with 13% repeat surgeries for mesh exposure in this 7-year period (Table 8, “Appendix 4” ). Ten patients (45%) with exposure experienced no symptoms; 12 patients (55%) reported pain. In 77% (17 patients), exposure was <1 cm (Addendum, Table 5, "Appendix 1” ). In the native tissue group, four patients (6%) had exposure: two due to mesh placement after the index surgery, one due to a Prolene suture used for sacrospinous ligament fixation and one after midurethral sling inserted after index surgery.

Data on pain and dyspareunia assessed by interview, self-completed questionnaires, and during gynecological examinations are shown in Table 8, “Appendix 4” . Estimates of patient self-reported pain scores (UDI) with and without multiple imputation were not significantly different between groups (Table 4). Per-protocol analysis did not reveal a significant difference either between mesh or native tissue repair (Table 4). Provoked pain during gynecological examination, however, was significantly more prevalent in the mesh group (Table 8, “Appendix 4” ).

**Table 4:** Pain at 7 years

	Mesh	Native tissue	Risk difference (95%CI)
Indicated on questionnaire (intention-to-treat analysis)			
Questionnaires (*n*)	66	72	
Pain: yes	26 (39)	36 (50)	−11 (−27, 6)
• UDI pain score	16.2 (16.2, 16.2)	20.2 (18.6, 21.8)	−4.1 (−11.3, 3.1)
• Estimated pain score after MI	17.8 (13.0, 22.6)	18.9 (14.6, 23.3)	−1.1 (−7.5, 5.2)
Having sexual intercourse	30 (46)	30 (42)	4 (−13, 20)
Indicated on questionnaire (per-protocol analysis)			
Questionnaires (*n*)	72	66	
Pain: yes	29 (40)	33 (50)	−10 (−26, 7)

Data presented as* n* (%) or mean (95% CI)

*MI* multiple Imputation,* UDI pain score* Urogenital Distress Inventory, range 0–100,* CI* confidence interval

At 7 years, 9 native tissue patients received mesh: 8 were transvaginal mesh (TVM), of whom 6 completed the questionnaire

Changes in health-related QoL scores between baseline and 7 years are shown in Table 6, “Appendix 2”; neither group showed significant changes over time in sexual function measured with the PISQ-12.

## Discussion

The long-term follow-up of this multicenter RCT showed similar composite success rates 7 years after TVM or native tissue repair in women with recurrent POP. Women treated with mesh often developed POP in a non-mesh-treated vaginal compartment with the need for repeat surgery. In contrast, after native tissue repair, significantly more recurrences were seen in the treated compartments. This phenomenon has been reported for the 1-year follow-up [18, 19].

Compared with a composite success of 84% at 5-year follow-up in a French cohort study using the same mesh kit, composite success of 53% in our mesh group was considerably lower [20]. In the French study, all patients received total vaginal mesh that treated all three vaginal compartments. Subanalysis of total mesh implantations in our study showed a composite success rate of 73% at 7 years, comparable with French results. One should consider, however, that total vaginal mesh implants are associated with a significantly higher risk of mesh exposure [21]. A remarkable finding in our study was the significant decrease between post hoc calculated composite success rates at 1 year and composite success rates at 7 years in both treatment groups. Increasing failure rates have also been reported 7 years after abdominal sacrocolpopexy [22].

For the long-term follow-up, we used a primary outcome different from that used in the index study. Barber et al. demonstrated in 2009 that patient impression of improvement was associated with absence of vaginal bulge symptoms [16]. The use of a composite outcome (combining anatomic and functional success, in the absence of retreatment) is therefore considered clinically more relevant and is in accordance with International Urogynecological Association and International Continence Society (IUGA–ICS) recommendations [16, 23].

The mesh exposure rate increased substantially over time, from 17% at 1 year to 42% at 7 years [8] There are a number of possible explanations. The mesh kit used consisted of relatively large pieces of nonabsorbable mesh (anterior 10 × 13 cm, posterior 7 × 16 cm) with relatively high weight of 45 g/m^2^. The total amount and weight of implanted mesh are correlated with the risk of exposure [21, 24–26]. Furthermore, 22 surgeons performed index surgeries, and although they were all considered experienced, they had their own learning curves and levels of experience [21]. An increase in exposure rates over the years has also been described in the extended follow-up of abdominal sacrocolpopexy [22].

In the media and according to lawsuits, there are numerous reports of patients with chronic pelvic pain and dyspareunia after mesh insertion [27]. We used various tools to assess pain and dyspareunia: patient self-completed questionnaires, doctor interviews, and VAS scores. We observed nonsignificant differences in ratings of pain and dyspareunia between groups. We hypothesize that repeat POP surgery itself may cause pain, and pain may not be solely caused by the mesh.

Change in primary outcome is a limitation of this study, since the index study was powered on anatomic success and not on subjective outcomes, which probably would have needed a larger sample size. Loss of participants in long-term follow-up studies is inevitable, particularly when considering the advanced mean age of the target group. Restricting the analysis to patients who completed the 7-year follow-up provides valid results if missing data are completely random; otherwise, it generates potentially biased results. Use of the multiple imputation technique, by which imputations of missing values are based on other known patient characteristics, leads to unbiased results with correct standard errors [28]. However, although this approach removes bias, it does not add power to the study. Data should therefore be interpreted with caution, since the study lacks power on negative outcomes. Furthermore, continuous negative media attention may have influenced the way participants responded to the questionnaires.

Although the mesh kit was withdrawn from the market in 2013, long-term data are still relevant to many patients, their doctors, and other stakeholders, e.g., health care inspectorates. According to sales information by Johnson & Johnson, >220,000 mesh kits were sold worldwide, in addition to comparable kits sold by other companies.

Strengths of this study are the design (multicenter RCT), systematic long-term follow-up, physical exam by one independent observer (blinded to the index procedures), and the high response rate. Furthermore, the extensive assessment of pain and dyspareunia with data at both baseline and 7 years after index surgery is unique. The 2006 American College of Obstetricians and Gynecologists Committee Opinion on Ethical Guidelines in Innovative Practice state that: “without adequate data on risks and benefits of new treatments, patients are unable to provide truly informed consent” [29]. Our long-term evaluation may contribute to these guidelines and offer support when counseling patients with recurrent POP, specifically regarding long-term risks of recurrence, pain, and dyspareunia following vaginal mesh kit surgery for POP.

Findings in this study strengthen the necessity of long-term observations on safety and efficacy when surgical devices are used in POP surgery. In every woman with recurrent POP, a native tissue repair can be reconsidered, preferably based on a prediction model [30]. Only if native tissue repair is expected to be inferior should a smaller piece of lightweight vaginal mesh or an abdominal mesh procedure be considered, but only if performed by experienced surgeons [31]. It is of utmost importance to counsel every woman planning to undergo any kind of POP surgery on the risks of pain and dyspareunia, particularly in case of repeat surgery.

## Appendix 1

**Table 5:** Addendum. Classification of complications by category and grades of pain at clinical visit 7 years following surgery

		Mesh (*n* = 53)		Native tissue (*n* = 67)		Risk difference (95% CI)
Any complication		40 (75)		39 (58)		17.3%* (0.7, 33.8)
Category	Grades of pain	Asymptomatic	Symptomatic	Asymptomatic	Symptomatic	
		12 (23)	28 (53)	5 (8)	34 (51)	
1		2 (4)	16 (30)	2 (3)	33 (49)	
	a	2		2		
	b		7		5	
	c		6		9	
	d		1		2	
	e		2		17	
2		8 (15)	9 (17)	2 (3)	1 (2)	
	a	8		2		
	b		2			
	c		3			
	d					
	e		4		1	
3		2 (4)	3 (6)	1 (2)	0 (0)	
	a	2		1		
	b		2			
	c		1			
	d					
	e					

Data presented as numbers (%)

Category: (1) Vaginal, no epithelial separation; includes prominence or excessive scarring, (2) vaginal epithelial separation ≤1 cm, (3) vaginal epithelial separation ≥1 cm

Grades of pain: (a) Asymptomatic or no pain, (b) provoked pain only (during vaginal examination), (c) pain during sexual intercourse, (d) pain during physical activities, (e) spontaneous pain

*This difference would be statistically significant if tested at a significance level of 0.05

## Appendix 2

**Table 6:** Changes in health-related quality of life and sexual function between baseline and 7 years

		Mesh (*n* 66)	Native tissue (*n* 76)	Difference (95% CI)
Mean change in UDI domain scores				
UDI	Overactive bladder	−6.3 ± 25.0	−3.4 ± 27.5	2.9 (−6.8, 12.6)
	Incontinence	3.3 ± 26.0	1.3 ± 27.0	−2.0 (−11.5, 7.5)
	Obstructive micturition	−6.0 ± 27.6	−4.8 ± 29.9	1.2 (−9.0, 11.5)
	Pain	−7.8 ± 24.4	−12.4 ± 31.2	−4.6 (−14.8, 5.5)
	Genital prolapse	−41.5 ± 31.8	−41.9 ± 37.3	−0.4 (−12.7, 11.9)
Mean change in DDI domain scores				
DDI	Constipation	−0.3 ± 17.8	−2.4 ± 16.8	−2.1 (−8.4, 4.2)
	Obstructive defecation	2.1 ± 18.4	−5.2 ± 19.1	−7.3 (−14.2, 0.5)
	Pain	−5.8 ± 17.6	−0.8 ± 19.6	5.0 (−1.5, 11.7)
	Fecal incontinence	4.5 ± 20.5	1.3 ± 26.5	−3.2 (−11.7, 5.3)
	Flatus	2.2 ± 37.8	2.0 ± 36.0	−0.2 (−13.2, 12.8)
Mean change in IIQ scores				
IIQ	Physical	−10.6 ± 26.4	−13.5 ± 26.4	−2.9 (−12.4, 6.5)
	Mobility	−5.6 ± 24.3	−9.3 ± 22.9	−3.7 (−11.8, 4.6)
	Social	−5.0 ± 19.6	−8.4 ± 22.6	−3.4 (−11.2, 4.6)
	Embarrassment	−0.9 ± 20.6	−0.5 ± 23.4	0.4 (−7.6, 8.4)
	Emotional	−3.6 ± 25.4	−4.8 ± 24.3	−1.2 (−10.0, 7.6)
Mean change in PISQ-12 scores		0.0 ± 4.2	2.9 ± 6.8	−2.9 (−6.8, 1.1)

Data presented as mean (domain baseline scores minus corresponding 7-year domain scores) ± standard deviation (SD). Negative scores in change reflect reduction in bother and improved quality of life compared with baseline

*UDI* Urogenital Distress Inventory,* DDI* Defecatory Distress Inventory,* IIQ* Incontinence Impact Questionnaire (0–00),* PISQ-12* Prolapse and Incontinence Sexual Questionnaire short form (positive change from baseline indicates better sexual function).

## Appendix 3

**Table 7:** Results per vaginal compartment at 7 years

Results per vaginal compartment			
Anterior compartment (level II treatment)	Anterior mesh	Anterior colporrhaphy	
Reoperation POP anterior compartment	0/32 (0)	3/39 (8)	−8 (−16, 1)
POP ≥ stage II anterior compartment	8/32 (25)	25/39 (64)	−39* (−60, −18)
Reoperation POP and/or POP ≥ stage II	8/32 (25)	27/39 (69)	−44* (−65, −23)
POP > hymen anterior compartment	3/32 (9)	8/39 (20)	−11 (−27, 5)
Reoperation POP and/or POP > hymen	3/32 (9)	11/39 (28)	−19* (−36, −2)
Composite success			
• Anterior mesh only (level II)	14/24 (58)		
• Anterior & total mesh (level II + I)	22/35 (63)	18/40 (45)	18 (−4, 40)
Apical compartment (level I treatment)	Apical mesh	Native tissue	
Reoperation for POP apical compartment	0/11 (0)	3/34 (9)	−9* (−11, −4)
POP ≥ II apical compartment	1/11 (9)	1/34 (3)	6 (−12, 24)
Reoperation POP and/or POP-Q ≥ II	1/11 (9)	4/34 (12)	−3 (−23, 18)
POP apical compartment > hymen	1/11 (9)	1/34 (3)	6 (−12, 24)
Reoperation POP and/or POP > hymen	1/11 (9)	3/34 (9)	0 (−19, 20)
Composite success	8/11 (73)	16/36 (44)	29 (−3, 60)
Posterior compartment (level II treatment)	Mesh	Colporrhaphy	
Reoperation POP posterior compartment	1/32 (3)	5/45 (11)	−8 (−19, 3)
POP ≥ II posterior compartment	4/32 (12)	13/43 (30)	−18 (−36, 0)
Reoperation POP and/or POP-Q ≥ II	4/32 (12)	18/45 (40)	−28* (−46, −9)
POP posterior compartment > hymen	1/32 (3)	1/43 (2)	1 (−7, 8)
Reoperation POP and/or POP > hymen	2/32 (6)	6/45 (13)	−7 (−20, 6)
Composite success			
• Posterior mesh only (level II)	9/23 (39)		
• Posterior and total mesh (level II + I)	17/34 (50)	25/45 (56)	−6 (−28, 17)

Data presented as* n* (%),* n* (%, 95% CI), or % (95% CI)

* POP* pelvic organ prolapse

*This difference would be statistically significant if tested at a significance level of 0.05

## Appendix 4

**Table 8:** Adverse events at 7 years

	Mesh	Native tissue	Risk difference (95% CI)
De novo SUI	12/62 (19)	8/66 (12)	7 (−5, 20)
Graft-related adverse events^a^			
• Exposure	22/53 (42)	4/67 (6)^b^	36* (21, 50)
• Retraction mesh during examination	17/53 (32)	2/67 (3)	29* (16, 42)
• Reoperation for complication	8/53 (15)	4/68 (6)	9 (−2, 20)
• Reoperation for exposure	7/53 (13)	0/68 (0)	13* (4, 22)
Pain and dyspareunia			
Indicated at the outpatient visit			
Pain	8 (15)	20 (30)	−15 (−30, 0)
Dyspareunia	14 (26)	15 (22)	4 (−12, 20)
Pain and/or dyspareunia	17 (32)	29 (43)	−11 (−28, 6)
Location of pain			
• Vulvar	1 (6)	2 (7)	−1 (−16, 14)
• Vaginal	1 (6)	5 (17)	−11 (−29, 6)
• Lower abdomen	4 (24)	8 (28)	−4 (−30, 22)
• Lower back	0 (0)	4 (14)	−14 (−26, 0)
• Other	11 (65)	10 (34)	29* (2, 59)
VAS (spontaneous pain)	1.4 ± 2.3	2.0 ± 2.3	−0.6 (−2.0, 0.8)
VAS (during physical activity)	2.0 ± 3.1	2.3 ± 2.5	−0.3 (−2.0, 1.4)
VAS (during sexual intercourse)	4.6 ± 3.6	2.7 ± 3.3	1.9 (−0.2, 4.0)
During gynecological examination			
Provoked pain	24 (45)	16 (24)	21* (5, 38)
• Apical	12 (50)	11 (69)	−19 (−49, 12)
• Mesh arms (distal/proximal)	8 (33)	2 (12)	21 (−4, 46)
• Deep ligament	1 (4)	1 (6)	−2 (−16, 12)
• Other	3 (12)	2 (12)	0 (−21, 21)
VAS (patient)	5.2 ± 2.1	5.3 ± 2.2	−0.1 (−1.5, 1.3)
VAS (subjective impression of examiner)	5.3 ± 2.2	4.8 ± 2.1	0.5 (−0.9, 1.9)
Indicated on questionnaire			
Dyspareunia during sexual intercourse	13/64 (20)	12/72 (17)	3 (−9, 17)
• Somewhat	5 (38)	6 (50)	−12 (−50, 27)
• Moderately	4 (31)	4 (33)	−2 (−39, 34)
• Quite a bit	4 (31)	2 (17)	14 (−19, 47)

Data presented as numbers (%) or mean ±  standard deviation (SD) and mean (95% CI)

* SUI* stress urinary incontinence,* VAS* visual analog scale, * CI* confidence interval

^a^Cumulative up to 7 years; more details on graft-related adverse events and morbidity are shown in the “Addendum”

^b^Exposure of Prolene thread in sacrospinous ligament fixation (1), exposure after midurethral sling for incontinence (1), exposures of mesh inserted for POP recurrence after index surgery (2)

*This difference would be statistically significant if tested at a significance level of 0.05
